# Effect of the Esterification of Starch with a Mixture of Carboxylic Acids from *Yarrowia lipolitica* Fermentation Broth on Its Selected Properties

**DOI:** 10.3390/polym12061383

**Published:** 2020-06-19

**Authors:** Ewa Zdybel, Tomasz Zięba, Ewa Tomaszewska-Ciosk, Waldemar Rymowicz

**Affiliations:** 1 Department of Food Storage and Technology, Faculty of Food Science, Wroclaw University of Environmental and Life Sciences, 37 Chełmońskiego Street, 51-630 Wrocław, Poland; tomasz.zieba@upwr.edu.pl (T.Z.); ewa.tomaszewska-ciosk@upwr.edu.pl (E.T.-C.); 2 Department of Biotechnology and Food Microbiology, Faculty of Food Science, Wroclaw University of Environmental and Life Sciences, 37 Chełmońskiego Street, 51-630 Wrocław, Poland; waldemar.rymowicz@upwr.edu.pl

**Keywords:** potato starch, esterification, *Yarrowia lipolitica* yeast, fermentation broth

## Abstract

Potato starch was esterified with carboxylic acids contained in the fermentation broth from *Yarrowia lipolitica* yeast production. Various acid concentrations and various roasting temperatures were used to determine effects of process conditions on ester properties, including the number of acid residues attached to starch chains, starch susceptibility to amylolysis, and thermal characteristics of starch phase transitions. Study results demonstrated the effect of both the composition and the dose of the fermentation broth and of roasting temperature of starch on the number of acid residues attached to starch chains. Citric acid was more susceptible to esterification with starch (DS = 5.65%) compared to the α-ketoglutaric acid (DS = 0.12%). In the case of the latter, a higher degree of substitution was determined in the esters produced at higher roasting temperatures. The lowest digestibility (RS = 20%) was demonstrated for the starch esters with the highest degree of substitution with citric acid, whereas all starch esters showed decreased values of the thermal characteristics of pasting.

## 1. Introduction

Starch modification through esterification involves starch chain reactions with inorganic and organic acids, salts of inorganic acids, as well as anhydrides or chlorides of organic acids. During the esterification process, acid groups linked by ester bonds with carbon atoms at positions 2, 3, and 6 of the anhydroglucose rings attach to the molecules of amylose and amylopectin [1]. Production methods and properties of starch esters have been extensively described. For instance, Zięba and co-workers esterified starch in a water suspension with acetic anhydride in an alkaline medium. This method allowed obtaining a degree of substitution (DS) at ca. 0.1 [2]. A higher degree of esterification with acetic acid (DS = 0.27) was obtained by these authors using the same method but with retrograded potato starch as a substrate [3]. In turn, Zhao et al. [4] have proved that the microwave pretreated starch samples showed higher acetylation degree compared to the microwave untreated samples. Kapelko-Żeberska et al. [5] described the production process of starch citrates via potato starch roasting using a citric acid solution, which allowed them to obtain preparations with the content of citric acid residues approximating 10%. Esters of starch with succinic acid were described by Zdybel et al. [6], who employed various esterification methods and obtained the highest degree of substitution (DS = 0.07) for roasted potato starch that had earlier been extruded and saturated with succinic acid anhydride. A higher degree of starch substitution with succinic acid (DS = 1.7) was obtained by Chang et al. [7], who roasted porous starch saturated with a solution of succinic acid anhydride. In turn, Rudnik et al. [8] employed the method of reactive extrusion to produce starch succinates (DS = 0.6), whereas Bhosale et al. [9] esterified starch with succinic acid anhydride in an aqueous solution with controlled pH (DS = 0.02). Starch esterification with phosphoric acid was conducted by Passauer et al. [10], who obtained the degree of substitution approximating 0.25 in the case of potato starch, DS = 0.53 in the case of waxy starch, and DS = 0.65 in the case of high-amylose starch. Staroszczyk and Janas [11] used microwaves to synthesize silicated starch and obtained its DS. at 0.91. There are also many works describing starch esterification with fatty acids. For instance, Muliana et al. [12] synthesized starch with vinyl and methyl esters, and with fatty acid anhydrides under high-pressure conditions and in modified atmosphere, and achieved starch DS approximating 0.28. Likewise, Junistia et al. [13] used esters of higher fatty acids and produced maize starch esters with DS < 2.

Apart from ample descriptions of starch ester synthesis upon starch modification with homogenous reagents, the available literature provides some reports on starch esterification with mixtures of acids. Šubarić et al. [14] and Ačkar et al. [15] modified wheat starch using mixtures of succinic acid with acetic anhydride as well as azelaic acid with acetic anhydride, and achieved its DS = 0.148. In turn, by modifying barley starch with a mixture of acids, they [16] obtained DS = 0.08.

Ample research works indicate the feasibility of starch modification using various acids and methods. Most of them concern the use of pure reagents as esterifying agents, whereas only few describe starch modification with naturally-synthesized acids derived from natural sources, like agri-food waste, but not isolated from them. An interesting method of starch modification involves the use of the fermentation broth from *Yarrowia lipolityca* yeast production. This broth contains various naturally-synthesized acids whose ratios differ depending on yeast strain applied, substrate used for biomass production, and culture conditions [17,18,19,20].

Yeast of the species *Yarrowia lipolityca* are capable of absorbing carbon from agri-industrial wastes [21] and of converting these wastes into industrially-valuable metabolites, like citric acid, pyruvic acid, α-ketoglutaric acid, mannitol, and erythritol [17,18,19,20]. In addition, the biological methods of the production of these compounds are more effective, safer, and cheaper, compared to the chemical methods [22]. The feasibility of starch esterification using a mixture of organic acids contained in the fermentation broth represents an interesting research issue due to the innovative possibilities of managing fermentation broth wastes and of producing various starch preparations with different properties.

The possibility of starch modification with a mixture of organic acids contained in a fermentation broth was described in our previous paper [23]. However, it is still an innovative research issue also due to a novel direction of managing fermentation broth waste. When modifying starch for practical purposes, one should remember that its properties depend on its botanical origin [9,24], reagent type [1,14,15], degree of substitution [2,25], and process conditions [5,6]. The production of a modified starch preparation with tailored properties requires a number of experiments to be conducted to optimize the modification procedure.

This study aimed to determine the effect of the composition and dose of the fermentation broth left after *Yarrowia lipolitica* yeast production and temperature of starch roasting on selected properties of starch esters produced by starch chain esterification with carboxylic acids from the broth.

## 2. Materials and Methods

### 2.1. Materials

Potato starch produced by PEPEES Niechlów in 2018 was modified with various doses of a mixture of carboxylic acids from the fermentation broth of *Yarrowia lipolitica* yeast. Experiments were carried out with two culture broths having various concentrations of organic acids, which were obtained from the Department of Food Biotechnology and Microbiology of the Wrocław University of Life Sciences. Their composition was as follows:

Broth A—α-ketoglutaric acid 60 g/L; citric acid 18 g/L; pyruvic acid 1 g/L.

Broth B—α-ketoglutaric acid 49 g/L; citric acid 46 g/L; pyruvic acid 1.5 g/L.

### 2.2. Production of Starch Preparations

Starch was modified according to the method for starch citrate production described by Kapelko-Żeberska et al. [5] and Zdybel et al. [23], in two parallel series.

Series I: Native potato starch was mixed with the culture broth. Broth doses were adjusted so as to obtain 15, 20, 25, 30, 35, and 40 g of the acids from the broth in 100 g starch dry matter. The mixtures were air-dried at 40 °C for 24 h, then cooled and ground. Next, the mixtures were dried, ground, and roasted in a convective dryer (Memmert, Schwabach, Germany) at 130 °C for 3 h. Afterward, the roasted samples were rinsed with portions of 60% ethyl alcohol (Honeywell, Seelze, Germany) at an alcohol to sample ratio of 4:1 to remove the excess of the reagent. Then, the samples were rinsed ten times, poured with 60% ethanol and left covered at a room temperature for 24 h. The rinsing cycle was repeated three times. The blank sample was prepared acc. to the analogous procedure but without broth addition.

Series II: Starch preparations were produced as in series I, but the culture broth added contained 20 g of acid per 100 g of starch, and the dried mixtures were roasted at various temperatures: 120, 130, 140, or 150 °C.

### 2.3. Determination of the Degree of Substitution

The degree of starch substitution (DS) was expressed as percentage content (g/100 g) of acid residues in the modified starch preparation and determined according to the method described by Zdybel et al. [23].

Two grams of the starch preparation tested were mixed with 100 mL of 0.5 M NaOH (Chempur, Piekary Śląskie, Poland) and left at room temperature for 24 h with continuous stirring. After this time, 250 mL of concentrated ethyl alcohol (Honeywell, Seelze, Germany) were added and the mixture was left again at room temperature for 24 h. The solution was filtered and concentrated to a volume of 50 mL. The content of acid residues esterified with starch was determined by HPLC (Thermo Fisher, Wien, Austria). A Carbohydrate H + column coupled with a UV detector (λ = 210 nm) and a refractometric detector at 65 °C with a liquid phase (25 nM trifluoroacetic acid) flow rate of 0.6 mL min^−1^ were used. Compounds were identified based on chromatograms of pure chemical standards [26,27]. The determinations were carried out at the Faculty of Food Biotechnology and Microbiology at the Wrocław University of Environmental and Life Sciences. The analysis was carried out in triplicate.

### 2.4. Characteristics of Phase Transitions

The phase transition characteristics were performed with a DSC 822 differential scanning calorimeter (Mettler Toledo, Schwerzenbach, Switzerland). Ten milligram dry weight of the modified starch preparations were weighed into a 100-µL aluminum crucible. Then, 30 mg of water were added and the crucible was covered with a lid. The samples thus prepared were conditioned at room temperature for 30 min and then placed in a calorimetric chamber at 25 °C and heated to 100 °C at a heating rate of 4 °C/min. The temperatures of onset, maximum, and end of the phase transformation, and the specific heat of phase transformation were read from the thermal curve obtained. The analysis was carried out in triplicate [23,25].

### 2.5. Susceptibility of Starch Esters to the Activity of Amylolytic Enzymes

An aqueous suspension of the modified starch preparations (0.72 g of preparation per 100 mL of solution) was prepared, which was diluted twice with an acetate buffer (pH = 4.3). The mixtures were then placed in a 37 °C water bath, and enzymes (α-amylases and glucoamylases) were added (Sigma, Steinheim, Germany). The amount of enzymes was adjusted to ensure complete saccharification after 20 min of hydrolysis of the native starch paste. Samples were incubated at 37 °C for 20 and 120 min. Afterward, a 1-mL sample was taken in which the enzymes were deactivated by placing the sample in a water bath at 100 °C. After cooling, the glucose content was determined using a glucose assay kit (BioSystem, Barcelona, Spain), which contains glucose oxidase and peroxidase. The concentration of the resulting colored complex was measured spectrophotometrically (Cecil, Cambridge, UK) at 500 nm and compared with a standard curve. The determined amount of glucose was used to calculate the percentage of rapidly digestible starch (RDS), slowly digestible starch (SDS), and amylolysis-resistant starch (RS) in the starch preparations. The analysis was carried out in triplicate [5,23].

### 2.6. Statistical Analysis

Study results were subjected to a statistical analysis using Statistica 13.1 software package (StatSoft Polska, Kraków, Polska). Two-way analysis of variance was conducted. The significance of differences between mean values was determined with Duncan’s test at a significance level of α = 0.05.

## 3. Results and Discussion

**Figure 1:**
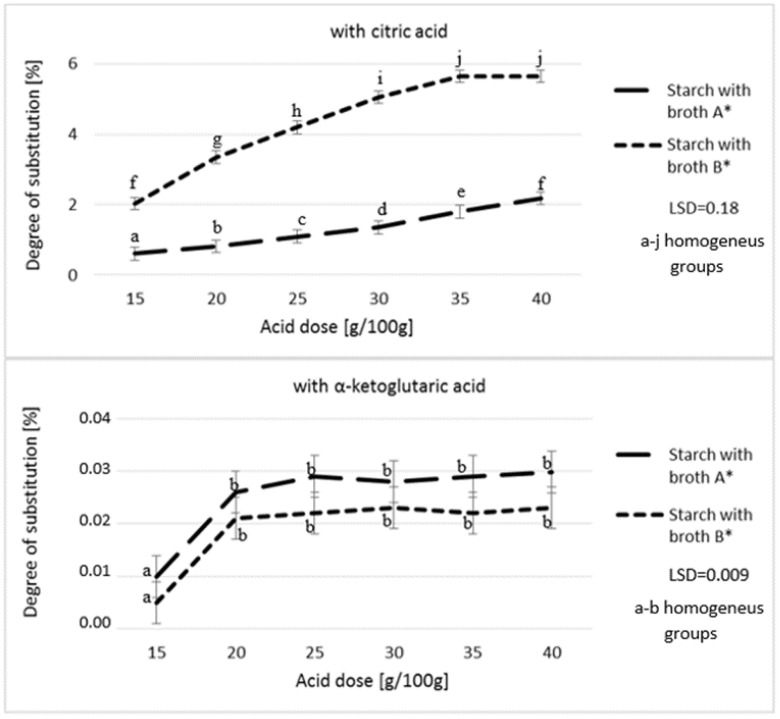
Effect of acid dose on the degree of starch substitution. * Broth A—α-ketoglutaric acid 60 g/L; citric acid 18 g/L; pyruvic acid 1 g/L; Broth B—α-ketoglutaric acid 49 g/L; citric acid 46 g/L; pyruvic acid 1.5 g/L.

**Figure 2:**
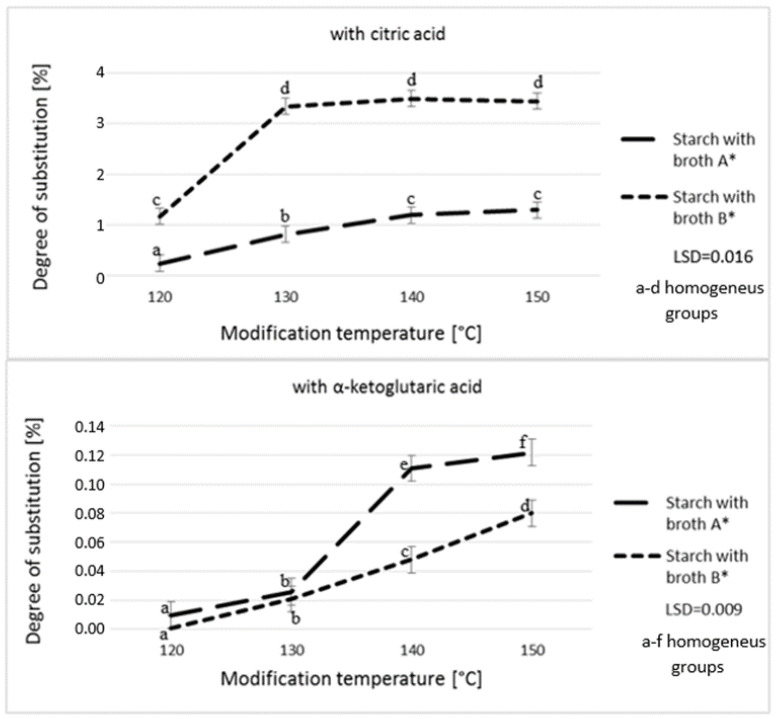
Effect of modification temperature on the degree of starch substitution. * Broth A—α-ketoglutaric acid 60 g/L; citric acid 18 g/L; pyruvic acid 1 g/L; Broth B—α-ketoglutaric acid 49 g/L; citric acid 46 g/L; pyruvic acid 1.5 g/L.

Figure 1 and Figure 2 present the degree of starch substitution (DS) as affected by the type and dose of the fermentation broth and also by the roasting temperature used in the starch modification process. The DS was presented as the percentage content of grams of acid residues in 100 g of the modified preparations. The analysis of contents of citric acid and α-ketoglutaric acid in the modified starch preparations demonstrated even 200-fold higher content of citric acid compared to α-ketoglutaric acid. Citric acid is highly capable of attaching to starch chains, as proved in many works addressing starch citrates [5,28]. Attempts of producing starch esters with citric acid and α-ketoglutaric acid have already been described in literature [23]. Results of the present study confirm that citric acid contained in a mixture of acids impairs the possibility of starch ester formation with α-ketoglutaric acid. As reported by other authors [29,30], the amount of the citric acid attached increases along with its dose in the culture broth and is higher in the case of using the broth with its higher content compared to the broth with a higher content of α-ketoglutaric acid. In addition, it needs to be emphasized that no difference was found in citric acid content between esters produced using the two highest doses of the broth with a higher content of citric acid. This may indicate that increasing the citric acid dose would increase the DS only to a certain value, while successive increase in its dose would not increase the amount of starch citrate produced. A higher DS can, probably, be obtained by using other esterification methods [5,31,32].

A similar dependency was noted while analyzing starch substitution with α-ketoglutaric acid. Only the 20 g dose of acid mixture added to starch caused an increase in α-ketoglutaric acid content compared to the dose of 15 g. The successive increase in the dose of acids had no effect on the amount of α-ketoglutaric attached to starch chains.

The analysis of the roasting temperature on starch substitution degree demonstrated the temperature of 130 °C to be optimal in the samples with a higher content if citric acid. The successive increase in roasting temperature had no effect on the amount of acid attached. In the case of the broth with a lower citric acid content, the roasting temperature of 140 °C resulted in a small increase in starch DS compared to the samples roasted at 130 °C. However, the successive temperature increase failed to ensure a statistically significant increase of the degree of substitution. As reported by other authors, the long-term roasting at a high temperature can decrease reaction dynamics due to the degradation of citric acid [30]. In turn, the higher roasting temperatures enabled obtaining a higher degree of starch substitution with α-ketoglutaric acid. In the case of both broths, a statistically higher degree of starch substitution with this acid was achieved at the roasting temperature of 150 °C. Roasting starch at the higher temperatures causes its thermolysis, transglucosidation, and repolymerization [33], therefore, a temperature of 150 °C seems to be optimal in the case of the reaction of starch chains with α-ketoglutaric acid.

None of the produced preparations contained pyruvic acid, despite its presence in the fermentation broth [23].

**Figure 3:**
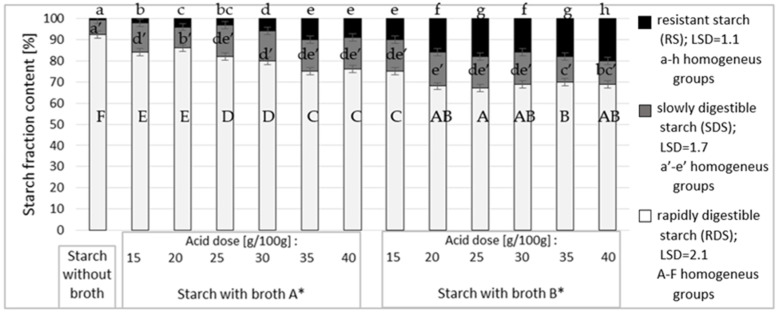
Effect of acid dose on the resistance of starch esters. * Broth A—α-ketoglutaric acid 60 g/L; citric acid 18 g/L; pyruvic acid 1 g/L; Broth B—α-ketoglutaric acid 49 g/L; citric acid 46 g/L; pyruvic acid 1.5 g/L.

Figure 3 shows the content of resistant starch in the modified starch preparations as affected by the dose and type of broth. To determine effects of the esterifying factors analyzed in the study, starch roasted without acids was used as a blank sample.

The roasted starch has small amounts of resistant starch (RS) and slowly-digestible starch (SDS), which was confirmed in works of other authors [23]. The reasons behind that should be searched in the unnatural bonds formed as a result of re-polymerization at the 2nd and 3rd atom of carbon, which are non-degradable in the process of enzymatic hydrolysis [33,34]. Literature data published so far corroborate the effect of citric acid attachment to starch chains on the increased starch resistance to the activity of amylolytic enzymes [5,35,36,37]. Results obtained in the present study demonstrate the lowest digestibility of the modified preparations having the highest degree of substitution with citric acid. Importantly, the preparations substituted with citric acid at DS = 3.3. showed no proportional increase in their resistance to the activity of amylolytic enzymes. Presumably, starch resistance is determined not only by the number of ester substituents but also by the site of their substitution. Zięba et al. [2] demonstrated that starch esterification at the 2nd and 3rd atom of carbon (neighboring to the 1,4-glycosidic bond being hydrolyzed) was largely responsible for the resistance of starch acetates.

**Figure 4:**
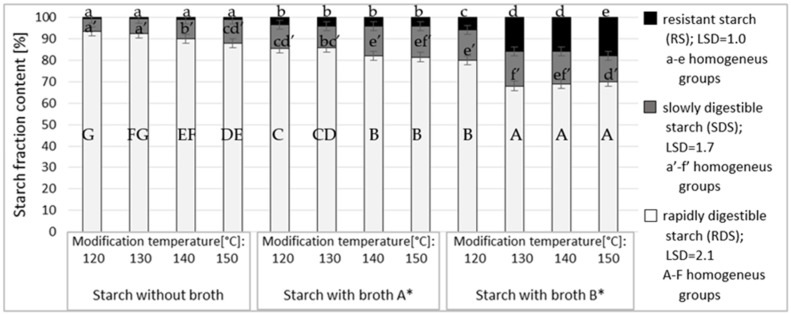
Effect of modification temperature on the resistance of starch esters. * Broth A—α-ketoglutaric acid 60 g/L;—citric acid 18 g/L; pyruvic acid 1 g/L. Broth B—α-ketoglutaric acid 49 g/L; citric acid 46 g/L; pyruvic acid 1.5 g/L.

The analysis of the effect of roasting temperature on digestibility of starch esters produced with broth A (Figure 4) revealed the lowest digestibility for the preparations roasted at 140 and 150 °C. Among starch esters produced with broth B significantly higher digestibility was determined for these roasted at 120 °C. Among the starch preparations tested, the highest resistance was determined for these modified using the broth with a higher content of citric acid and these roasted at the temperature of 130 °C or higher. The analysis of study results allows concluding that the conditions used to produce starch esters and the applied doses of broth with various acid ratios affected the percentage of esters substitution with acids which, in turn, was reflected in their resistance. Since there were no samples of starch preparations substituted with α-ketoglutaric acid only, it was impossible to determine its effects on the resistance of the modified preparations. However, in our previous work [23], the preparation substituted only with this acid and having DS of ca. 0.03 showed no increase in resistance compared to the roasted starch.

Table 1 presents results of the thermal analysis. The thermal characteristics of pasting of roasted starch is determined by roasting temperature, starch type, and moisture content of starch [38]. In the case of the preparations produced using broth A, the lowest energy of phase transition and the lowest temperature of the onset, end, and the maximum temperature of phase transition were demonstrated for the esters produced with the highest dose of the broth. In the case of broth B, its addition at 30% and more caused a significant decrease in the heat of phase transition. These results confirmed the earlier discussed results of the degree of substitution. The preparations with the highest DS were characterized by the lowest temperatures of the onset, end, and maximum of transition, and by the highest values of phase transition energy. This dependency was also confirmed in other works [24,25,28,39,40].

In the case of starch esters produced at various roasting temperatures (Table 2), both the temperatures of phase transition and the heat of transition were observed to decrease along with an increasing temperature of roasting, which is consistent with literature data [5]. This decrease could be due to starch substitution [5,23] and degradation of its chains [41] that could occur during starch roasting at a high temperature in the presence of acids.

## 4. Conclusions

The study demonstrated the effect of both the composition and dose of culture broth as well as of roasting temperature on the number of acid residues attached to starch chains. Citric acid was more susceptible to the esterification with starch compared to the α-ketoglutaric acid. At acid doses below 35%, the degree of starch substitution with citric acid increased along with increasing amounts of acids used. In turn the degree of starch substitution with α-ketoglutaric acid showed no changes at acids doses above 20%. In the case of α-ketoglutaric acid, a higher degree of starch substitution was determined in the preparations produced at the higher roasting temperatures. Lower digestibility was found for the preparations having the highest degree of substitution with citric acid. All starch esters produced showed decreased values of the thermal characteristics of pasting.

## Figures and Tables

**Table 1:** Thermal analysis of starch esters as affected by acid dose.

Starch Preparation	Acid Dose (g/100 g)	Specific Heat of Phase Transition (J/g)	Temperature of Onset of Phase Transition (°C)	Temperature of Maximum of Phase Transition (°C)	Temperature of End of Phase Transition (°C)
Starch without broth	0	11.62 ± 0.25 ^k^	54.83 ± 0.64 ^g^	61.27 ± 0.49 ^i^	69.87 ± 0.58 ^h^
Starch with broth A	15	9.97 ± 0.20 ^j^	48.66 ± 0.52 ^f^	56.00 ± 0.42 ^h^	68.02 ± 0.58 ^g^
	20	8.64 ± 0.21 ^i^	43.91 ± 0.52 ^e^	53.67 ± 0.48 ^g^	65.90 ± 0.60 ^f^
	25	7.80 ± 0.21 ^h^	41.84 ± 0.48 ^bc^	51.02 ± 0.30 ^f^	62.63 ± 0.46 ^e^
	30	7.02 ± 0.19 ^g^	41.64 ± 0.41 ^b^	50.84 ± 0.33 ^ef^	62.80 ± 0.42 ^e^
	35	6.92 ± 0.29 ^g^	41.28 ± 0.53 ^ab^	50.42 ± 0.48 ^de^	62.86 ± 0.58 ^e^
	40	5.67 ± 0.40 ^f^	40.88 ± 0.55 ^a^	49.94 ± 0.50 ^cd^	61.52 ± 0.55 ^d^
Starch with broth B	15	5.18 ± 0.22 ^e^	43.95 ± 0.40 ^e^	53.79 ± 0.33 ^g^	63.17 ± 0.39 ^e^
	20	3.89 ± 0.31 ^d^	42.68 ± 0.61 ^d^	49.82 ± 0.58 ^c^	60.98 ± 0.58 ^d^
	25	3.01 ± 0.38 ^c^	42.64 ± 0.68 ^d^	48.02 ± 0.52 ^b^	58.14 ± 0.58 ^c^
	30	1.62 ± 0.19 ^b^	42.44 ± 0.45 ^cd^	48.28 ± 0.33 ^b^	57.23 ± 0.40 ^b^
	35	1.29 ± 0.19 ^a^	41.74 ± 0.47 ^b^	47.14 ± 0.33 ^a^	55.20 ± 0.45 ^a^
	40	1.29 ± 0.21 ^a^	41.37 ± 0.47 ^ab^	47.21 ± 0.40 ^a^	55.36 ± 0.45 ^a^
LSD		0.32	0.64	0.50	0.62

^a–k^-homogeneus groups.

**Table 2:** Thermal analysis of starch esters as affected by modification temperature.

Starch Preparation	Modification Temperature (°C)	Specific Heat of Phase Transition (J/g)	Temperature of Onset of Phase Transition (°C)	Temperature of Maximum of Phase Transition (°C)	Temperature of End of Phase Transition (°C)
Starch without broth	120	11.72 ± 0.28 ^h^	54.79 ± 0.60 ^g^	61.30 ± 0.52 ^g^	70.10 ± 0.58 ^j^
	130	11.62 ± 0.25 ^h^	54.83 ± 0.64 ^g^	61.27 ± 0.49 ^g^	69.87 ± 0.58 ^ij^
	140	10.97 ± 0.36 ^g^	54.46 ± 0.58 ^g^	61.13 ± 0.44 ^fg^	69.38 ± 0.45 ^hi^
	150	9.88 ± 0.34 ^f^	53.60 ± 0.55 ^f^	60.74 ± 0.40 ^f^	69.25 ± 0.48 ^h^
Starch with broth A	120	9.12 ± 0.22 ^e^	45.18 ± 0.50 ^e^	55.12 ± 0.46 ^e^	67.54 ± 0.56 ^g^
	130	8.64 ± 0.21 ^d^	43.91 ± 0.52 ^d^	53.67 ± 0.48 ^d^	65.90 ± 0.60 ^f^
	140	5.94 ± 0.40 ^c^	40.54 ± 0.56 ^b^	50.02 ± 0.48 ^b^	62.64 ± 0.55 ^d^
	150	5.89 ± 0.41 ^c^	40.12 ± 0.54 ^ab^	49.80 ± 0.42 ^b^	58.81 ± 0.54 ^b^
Starch with broth B	120	4.01 ± 0.30 ^b^	44.92 ± 0.60 ^e^	51.62 ± 0.59 ^c^	63.74 ± 0.58 ^e^
	130	3.89 ± 0.31 ^b^	42.68 ± 0.61 ^c^	49.82 ± 0.58 ^b^	60.98 ± 0.58 ^c^
	140	3.12 ± 0.38 ^a^	39.74 ± 0.56 ^a^	46.20 ± 0.56 ^a^	55.50 ± 0.60 ^a^
	150	2.74 ± 0.31 ^a^	39.85 ± 0.60 ^a^	45.81 ± 0.58 ^a^	54.93 ± 0.60 ^a^
LSD		0.38	0.58	0.50	0.58

^a–j^-homogeneus groups.
